# Employment status and perceived health condition: longitudinal data from Italy

**DOI:** 10.1186/1471-2458-14-946

**Published:** 2014-09-12

**Authors:** Liliana Minelli, Claudia Pigini, Manuela Chiavarini, Francesco Bartolucci

**Affiliations:** Department of Experimental Medicine, University of Perugia, P.le Gambuli,1, 06122 Perugia, Italy; Department of Economics, University of Perugia, Via A. Pascoli 20, 06120 Perugia, Italy; Department of Economics and Social Sciences, Università Politecnica delle Marche, P.le Martelli 8, 60121 Ancona, Italy

**Keywords:** Self-reported health status, Employment status, Economic crisis, Fixed-effects ordered logit model

## Abstract

**Background:**

The considerable increase of non-standard labor contracts, unemployment and inactivity rates raises the question of whether job insecurity and the lack of job opportunities affect physical and mental well-being differently from being employed with an open-ended contract. In this paper we offer evidence on the relationship between self-reported health and the employment status in Italy using the Survey on Household Income and Wealth (SHIW); another aim is to investigate whether these potential inequalities have changed with the recent economic downturn (time period 2006-2010).

**Methods:**

We estimate an ordered logit model with self-reported health status (SRHS) as response variable based on a fixed-effects approach which has certain advantages with respect to the random-effects formulation: the fixed-effects nature of the model also allows us to solve the problems of incidental parameters and non-random selection of individuals into different labor market categories.

**Results:**

We find that temporary workers, first-job seekers and unemployed individuals are worse off than permanent employees, especially males, young workers, and those living in the center and south of Italy.

**Conclusion:**

Health inequalities between permanent workers and job seekers widen over time for male and young workers, and arise in the north of the country as well.

## Background

Job insecurity and the lack of work opportunities have characterized labor markets for the better part of the last decade. There has been a considerable increase of non-standard labor contracts, as opposed to permanent employment, alongside the increase in the unemployment rate, especially among young people. In Italy, for workers between 15 and 24 years of age, the share of temporary employment on total employment raised from 26.2% in 2000 to 52.9% in 2012 and the unemployment rate raised from 31.2% to 35.2% [1].

In this framework, one relevant question in applied works has been whether job insecurity and being out of the labor market affect the individuals’ well-being differently from being employed with a permanent contract. The available empirical evidence suggests that there is a negative relationship between being employed with a temporary contract and the individual health status and several contributions have also found a negative correlation between long-term unemployment, health condition, and mortality risk. Moreover, the intensity of this relationship has been proved to be strongly differentiated by gender and across countries, especially in relation with the institutional settings that characterize the labor markets.

The adverse effect of unemployment on health outcomes has been widely analyzed both in the medical and in the social-science literature. Job loss deteriorates health mainly for the experiencing of a stressful life event and through the risk of poverty and economic deprivation. Consequences range form the engagement in riskier health-related behaviors, leading to a higher incidence of cardio-vascular diseases especially among men, to a higher risk of depression and suicide^a^. Experiencing the job loss event has been found to be related to higher risks of a heart attack or stroke and with the risk of mental illness [2], to poorer self-rated health [3, 4] and ultimately with a higher mortality risk [5].

One relevant dimension in these studies is the length of unemployment, meaning that long-term unemployed are in worse physical and psychological health than those experiencing a short spell of unemployment [6]. More specifically, long-term unemployment has been found to be related to elevated all-cause mortality with a mortality risk increasing in the duration of unemployment [7]. Long–term unemployment is also associated with a greater incidence of suicide: in particular, the risk is greatest in the first five years, and persists at a lower level up to 16 years after the unemployment event [8].

While (long-term) unemployment has been steadily in the center of the investigation on social determinants of morbidity and mortality, a lively debate has been growing in the literature on precarious employment as an emerging socio-economic determinant of health [9, 10]. The empirical evidence has identified contract and income instability, along with worse working conditions including the psychological work environment, as the main channels describing the negative relationship between precarious employment and health outcomes. This literature is extensive and we only review some contributions among the most recent and closely related to our research question. For a more detailed review see [11].

Insecurity due to the contract duration has been found to substantially decrease the perceived health condition [12], to increase psychological distress [13] and even poor physical health [14]. Job insecurity has also contributed to an increase by 50% the level of depressive symptoms in the US [15]. Workers unsatisfied with their contract terms [16] report lower self-rated health and the insecurity due to contract uncertainty is such that transitions from unemployment to fixed-term employment does not increase the perceived health condition, at least in the short run [17]. In addition, self-rated health is sensitive to economic deprivation due to intermittent spells of employment, so that workers are unsatisfied with their material reward [18], and to the lack of benefits provided by a stronger employment protection [19]. Recently, cross-country analyses have also been carried out with the purpose of highlighting differences in the link between insecurity and poor health within Europe. The association between job insecurity and self-rated health has been studied using cross-sectional data from 16 European countries finding that precarious employment is not associated with poor health only in Belgium and Sweden [20]. Institution settings and labor market regulations can explain a significant part of cross-country differences [21]. In addition, precarious work in Bismarckian, Southern European, Anglo-Saxon, Eastern European, and East Asian countries is found to be associated with adverse physical health outcomes, such as musculoskeletal disorders and injuries [22].

Workers employed with a temporary contract often results in having to cope with worse working conditions. Among other determinants, the number of hours worked and the unpaid overtime work decrease self-rated health and psychological well-being [23]. In addition, temporary workers often experience disempowerment, especially in bargaining over wages and working hours, and little entitlement to workplace rights, that indicate a difficult psychological environment in the workplace. Analyses comprehensive of all these factors have used data from the Psycho-social Factors Survey carried out in 2004-2005 in Spain and employing the response variables form the *Employment Precariousness Scale* (EPRES) [24]. This variable comprises many dimensions of temporary employment (duration, economics deprivation, limited rights, vulnerability and defenselessness in the work place)^b^. Results confirm a negative association between job insecurity and poor mental health, especially stronger for women.

Recently, the literature has also focused on the evolution of the relationship between employment profile and health outcomes during periods of financial strain. Evidence for the US suggest that the relationship between working hours, change in type of contract and health after the 2008 economic crisis have no significant effect on health behaviors [25]. In contrast, the negative impact of unemployment, especially long-term, on mental health worsens significantly during the economic crisis in Spain [26].

Although the empirical works mentioned above have assessed the extent of this relationship for many OECD countries, the evidence for the case of Italy is indeed scarce. Only one contribution provides some empirical evidence for the Italian case [27]: the study employs the “Multiscopo” (Multiscope) survey issued by the Italian Institute of Statistics matched, by a simulation procedure, with some information (non–health related) on income present in SHIW. Youth temporary employment is analyzed for the years 2004/2005. It emerges that there is a negative relationship between psychological well-being, happiness and temporary employment especially for young male workers. However, their analysis is referred only to the pre-crisis period.

In this paper, we offer empirical evidence on the relationship between the self-assessed health status and the labor market position in Italy. In this regard, we compare the health conditions of temporary workers, first-job seekers and unemployed individuals with those of workers with fixed income (permanent employees and self-employed workers). We use the question on Self Reported Health Status (SRHS) in the SHIW issued by the Bank of Italy. This issue is particularly interesting in the case of Italy, where the labor market is characterized by a strong dichotomy: on one side permanent workers benefit from a considerably high degree of protection, while on the other temporary workers are exposed to a high job insecurity and a very low degree of employment protection. In addition, workers experiencing spells of unemployment, whose health condition may deteriorate due to the economic deprivation, are often not adequately sustained by welfare interventions.

Since the survey comprises SRHS for the time span 2006–2010, we are able to investigate whether this relationship has changed with the recent economic downturn, testing whether inequalities in the health conditions across the employment profiles have exacerbated with the occurrence of the economic crisis. The financial situation of the country has led to substantial cuts in public expenditure and also in health care. However, growing inequalities in the health conditions between economically deprived categories and the financially stable ones would call for a greater presence of the public health care system.

We additionally investigate whether the relationship between employment profiles and health is different across the country, since the Italian health care system in is the hands of regional governments potentially leading to inequalities in health care provision [28]. Finally, we uncover whether inequalities have settled in specific demographic groups. We differentiate our analysis by age and gender, as the lack of job opportunities and the raising incidence of non-standard labor contracts have specifically targeted young and female workers.

## Methods

### Data source

We use the question on SRHS in the panel SHIW issued by the Bank of Italy. The SHIW dataset is freely available and it can be downloaded at the address http://www.bancaditalia.it/statistiche/indcamp/bilfait. For the longitudinal study, about 2,000 households are interviewed every two years. The sample design is based on two stages: first, about 400 municipalities with more than 20,000 inhabitants are sampled; secondly, households (the group of people living in the same house or temporarily not in the same house but related) are sampled and interviewed by CAPI (Computer-Assisted Personal Interviewing). The participation rate (interviews on contacted families) was around 41% in 2006, 55% in 2008, and 53% in 2010^c^.

SHIW includes the question about SRHS, available at the individual level, for 2006, 2008 and 2010. For the time window 2006-2010 we build an unbalanced longitudinal dataset of 59,294 observations for which SRHS is observed at least twice. We further restrict the sample to individuals between 15 and 64 years of age and, after dropping outliers and observations with missing values in the variables of interest, we end up with a sample of 37,782 observations.

SRHS, labeled SALUT in the questionnaire, is an ordered variable that takes values between 1 and 5 for increasing health status: it takes value 1 if the respondent answered his/her health is *very poor* in the year of the interview, it takes value 2 for *poor*, 3 for *fair*, 4 for *good*, and 5 for *excellent*. Self-rated health if often questioned as a measure of health outcomes as its subjective nature in household surveys may produce biased results. Nevertheless, it is widely used in empirical applications and it has been recognized to be a valid predictor of morbidity and mortality [29, 30] since the process of health assessment ultimately lies in the biological and psychological state of the individual [31].

The first row of Table 1 contains the sample frequencies for each category of SRHS and shows that the majority of the respondents declare they are in good or excellent health.

**Table 1:** **Sample descriptive statistics of self reported health status (%) by employment status, year, gender, age, area of residence, family income, family wealth, and regional unemployment rate**

	Very poor	Poor	Fair	Good	Excellent	Total
**Total**	0.25	1.63	7.86	54.26	36.00	100.00
**Employment status**						
Permanent	0.13	0.91	5.84	55.27	37.85	48.25
Temporary	0.07	1.20	7.51	54.23	36.99	7.72
First-job seekers	0.05	0.76	3.08	50.22	45.89	4.90
Unemployed	0.47	3.96	13.08	55.60	26.89	3.95
Out of labor force	0.46	2.57	10.79	53.29	32.89	35.18
**Year**						
2006	0.19	1.66	8.04	54.43	35.68	33.23
2008	0.36	1.72	7.79	54.11	36.03	33.35
2010	0.21	1.50	7.75	54.25	36.30	32.43
**Gender**						
Female	0.23	1.68	8.36	55.53	34.18	50.76
Male	0.27	1.57	7.34	52.95	37.88	49.24
**Age**						
15–40	0.10	0.54	2.81	46.29	50.26	46.11
41–64	0.38	2.56	12.18	61.09	23.80	53.89
**Area of residence**						
North	0.21	1.30	6.79	48.33	43.36	43.13
Center	0.28	1.59	8.78	59.63	29.71	19.64
South	0.28	2.03	8.60	58.30	30.80	37.23
**Income**						
≤22,090	0.40	3.12	11.16	56.57	28.74	24.99
(22,090;33,490]	0.37	1.59	8.22	54.76	35.06	25.01
(33,490;47,850]	0.16	1.15	6.96	53.50	38.23	24.99
>47,850	0.07	0.65	5.09	52.22	41.97	25.01
**Wealth**						
≤40,500	0.30	2.35	9.03	54.88	33.44	24.99
(40,500;186,900]	0.35	2.06	8.42	55.53	33.63	25.01
(186,900;335,000]	0.26	1.14	7.21	53.12	38.27	25.00
>335,000	0.10	0.95	6.77	53.52	38.67	25.00
**Unemployment rate**						
≤4.3	0.23	1.43	7.51	48.16	42.67	25.12
(4.3;5.7]	0.24	1.50	6.87	54.39	37.00	25.30
(5.7;12.6]	0.23	1.50	9.03	55.95	33.30	24.53
>12.6	0.31	2.08	8.05	58.60	30.96	25.05

The employment status is a categorical variable that identifies five possible conditions in the labor market. In our empirical analysis, given the possible time-varying nature of the employment condition, we investigate the effect of experiencing each of these conditions one or more times on SRHS^d^. We label *permanent* dependent workers with an open–ended contract and self-employed individuals with a stable income^e^. The *temporary* category comprises job contracts such as apprenticeships, on-project jobs, and seasonal jobs^f^. *First-job seekers* are those who have being actively looking for their first job in the six months preceding the interview. The *unemployed* category refers to individuals who have being actively looking for a job in the six months preceding the interview and that would be available to work immediately. Finally, *out of labor force* embeds home makers, retired workers, and students. This last profile is only meant to serve as a residual category in the empirical analysis since its exclusion could result in a selection bias problem. However, with lack of further information, the causal effect of being out of the labor force on health cannot be identified since workers who stopped working due to their health limitations could be part of this category [32] ^g^.

The distribution of responses to SRHS by employment status does not exhibit major differences (see the top of Table 1). Nevertheless, the share of *first-job seekers* choosing the *excellent* category is higher than for the other classes and the *unemployed* subjects are those choosing the best category less frequently. Moreover, the share for responses *fair*, *poor*, and *very poor* is somewhat increasing from *permanent* to *out of labor force* with the exception of *first-job seekers*.

Table 1 also shows the respondents’ distribution by year, gender, age, and area of residence. The shape of the distribution of responses to SRHS has not dramatically changed with the occurrence of the economic crisis. Furthermore, Table 1 shows that there is a higher concentration of *good* responses for women compared to the higher share of *excellent* responses for male individuals. Finally, there is a clear difference in the distribution of SRHS between the North and the Rest of Italy: while in the North the majority of responses are equally shared between the *good* and the *excellent* category, for the rest of the country there is a tendency to concentrate responses on the *good* category.

We also present descriptive statistics for the household net income^h^, the household wealth and the regional unemployment rate^i^. Although they are not of primary interest in our analysis, income and wealth will be included (in thousands of Euros) in the model specification as proxy for economic deprivation, while the unemployment rate will give a measure of relative deprivation. For readability of the descriptive statistics, we categorized income, wealth, and regional unemployment rate on the basis of their quartiles: as expected, the relative distribution of the responses shifts towards higher categories of SRHS as income and wealth increase, while it progressively concentrates on the *good* category (leaving *excellent*) for increasing values of the local unemployment rate.

### Statistical analysis

The nature of SRHS is such that it needs to be modeled as an ordinal response variable and therefore non–linear models must be employed for estimation. In applied works, it is customary to consider the ordinal response variable as the results of a categorization mechanism that limits the observability of a latent continuous variable, as the health status can be. Therefore, we set up our model specification by defining the unobservable perceived health status as the latent continuous variable  for individual *i* at time *t* that is based on a linear combination of individual covariates collected in the column vector ***x***_*it*_ and unobservable characteristics represented by the random variables *α*_*i*_ and *ε*_*it*_:
1

In the above expression, the unobservable individual effect *α*_*i*_ is allowed to be correlated with ***x***_*it*_. The observable ordinal health status is related to the latent variable  by the following observational rule:
2

where the threshold parameters *τ*_*k*_, *k*=1,…,*K*, with *τ*_0_=-*∞* and *τ*_*K*_=*∞*, are strictly increasing and *K* is the number of response categories.

With cross-section data, the parameters ***β*** in (1) and the threshold parameters *τ*_*k*_ in (2) are estimated by Maximum Likelihood using the common ordered logit/probit models provided that the effects *α*_*i*_ are ruled out. However, the longitudinal structure of our dataset allows us to properly take into account the presence of the time-invariant individual unobserved heterogeneity effects *α*_*i*_.

With longitudinal data, one can either estimate a random-effects ordered probit/logit model or a fixed-effects ordered logit model. Choosing one or the other implies different assumptions on the effects *α*_*i*_. In order to consistently estimate a random-effects model, *α*_*i*_ needs to be independent of ***x***_*it*_ and assumptions on the joint distribution of *α*_*i*_ and *ε*_*it*_ must be made; in contrast, the fixed-effects ordered logit model does not require these assumptions as the individual time-invariant unobserved effects cancel out by a suitable transformation that is illustrated in the following. The fixed-effects model also provides an estimator robust to misspecification of the distribution of *α*_*i*_. Note that the estimator is less efficient than the one obtained with the random-effects model when the distributional assumptions on *α*_*i*_ arecorrect.

A fixed-effects ordered logit model is a better choice for health applications as it solves the problem of other possible sources of bias due to unobserved heterogeneity [4]. Most importantly, there is the issue of self-selection into employment categories [11, 33]. For instance, certain behaviors may result unfavorable for individuals’ “employability” and their health-related outcomes, however separately, at the same time [34]. By assuming that the propensity to engage in such behaviors is time-invariant in the time span considered, the bias, potentially generated by this spurious correlation, is eliminated if an ordered fixed-effects logit model is adopted.

The estimation of an ordered fixed-effects logit model can be reduced to the estimation of a fixed-effects binary logit model [35–37] once the *K* category responses have been transformed in *K* - 1 binary response variables categories. A consistent estimator can be obtained by conditioning each likelihood contribution corresponding to each of these dichotomizations on a sufficient statistic which is the sum of the individual outcomes over time. The parameters, associated with time-varying covariates, can then be estimated by Conditional Maximum Likelihood (CML).

By assuming that the the error terms *ε*_*it*_ are independent and identically distributed, standard logistically distributed conditionally on ***x***_*it*_ and *α*_*i*_, the probability that *y*_*it*_ takes value *k* for individual *i* at time *t* is
3

where *Λ* is the standard logistic cumulative distribution function. Following a standard notation [38], let  be the binary dependent variable defined as , that is the dichotomization of *y*_*it*_ at the cutoff *k*. It follows that *P*(*d*_*it*_=0)=*Λ*(*τ*_*k*+1_-***x****it*′***β***-*α*_*i*_) and *P*(*d*_*it*_=1)=1-*Λ*(*τ*_*k*+1_-***x****it*′***β***-*α*_*i*_) since . The sum over all elements of  is a sufficient statistic for *α*_*i*_, as in the binary model, and the thresholds *τ*_*k*_: by conditioning on  it can be shown that *α*_*i*_ can be eliminated^j^.

The conditional density of  is


where at the denominator there the set of all possible sequences of 0s and 1s for which the sum of *T* binary outcomes is equal to  that is .

The sample conditional log-likelihood is
4

The vector  resulting form the maximization of (4) is a consistent estimator of the parameters in (1). Only individuals with  and  contribute to the log-likelihood. In addition, the fixed-effect nature of the model is such that parameters associated with time-invariant covariates cannot be estimated because they are not identified.

Finally, we test the fixed-effects type against the random-effects specification of the model by means of the Hausman test [39]: under the null hypothesis of correct specification of the joint distribution of *α*_*i*_ and *ε*_*it*_, both the fixed-effects and the random-effects estimators are consistent but the latter is more efficient; under the alternative, only the fixed-effects estimator is consistent. However, the proposed test is not valid with heteroskedasticity and when time dummies are included in the model specification. Therefore, we estimate with a random-effects^k^ ordered logit model the auxiliary regression


where ***x***_*it*_ contains time-varying and time-invariant covariates and  are the time averages of all the time-varying regressors [40]. The Hausman test statistic is then computed as a Wald test for *H*_0_:**ξ**=**0** using the panel robust covariance matrix estimator.

## Results and discussion

Table 2 reports the estimation results of two models for the SRHS variable estimated by using the Fixed-Effects (FE) and the Random-Effects (RE) ordered logit models. The first model, M1, is estimated by using a specification that includes dummies for the employment condition and time dummies separately, while the specification of second model, M2, is augmented by their interaction terms. We include the household annual income and wealth in order to control for the effect of economic deprivation and we add their quadratic terms to capture the possible concave relationship with these covariates. Moreover, the regional unemployment rate serves as a proxy of relative deprivation. In addition, to control for aging effects, we include the age of the individual and its square in the RE model and only the age square in the FE model to avoid collinearity with the time dummies.

**Table 2:** **Fixed-effects (FE) and random-effects (RE) ordered Logit models for self reported health status**

	M1		M2	
	FE	RE	FE	RE
	coeff. (s.e.)	coeff. (s.e.)	coeff. (s.e.)	coeff. (s.e.)
Ref: Permanent				
Temporary	**-0.147** (0.100)	**-0.400** (0.058)	-0.159 (0.159)	**-0.455** (0.095)
First-job seekers	**-0.435** (0.139)	**-0.359** (0.077)	**-0.459** (0.195)	**-0.272** (0.127)
Unemployed	**-0.317** (0.150)	**-0.710** (0.086)	**-0.366** (0.237)	**-0.820** (0.143)
Out of LF	**-0.300** (0.091)	**-0.182** (0.039)	**-0.305** (0.091)	**-0.183** (0.039)
2008	-0.034 (0.043)	**0.075** (0.032)	-0.057 (0.047)	**0.066** (0.035)
2010	-0.031 (0.085)	**0.326** (0.036)	-0.012 (0.090)	**0.324** (0.039)
Fam. Income	0.038 (0.031)	**0.136** (0.011)	0.039 (0.031)	**0.136** (0.011)
Fam. Income sq.	**-0.384** (0.129)	**-0.267** (0.033)	**-0.390** (0.131)	**-0.267** (0.033)
Fam. Wealth	**0.050** (0.013)	**0.016** (0.005)	**0.050** (0.013)	**0.015** (0.005)
Fam. Wealth sq.	**-0.016** (0.005)	**-0.006** (0.003)	**-0.016** (0.005)	**-0.006** (0.003)
Unemp. rate	**0.095** (0.039)	**-0.060** (0.005)	**0.094** (0.040)	**-0.060** (0.005)
Age		**-0.015** (0.004)		**-0.015** (0.004)
Age ^2^	**-0.074** (0.008)	**-0.086** (0.004)	**-0.075** (0.008)	**-0.086** (0.004)
Temp. × 2008			0.075 (0.138)	0.060 (0.126)
Temp. × 2010			-0.046 (0.198)	0.101 (0.127)
FJ seek. × 2008			0.164 (0.205)	-0.024 (0.162)
FJ seek. × 2010			-0.104 (0.232)	-0.227 (0.160)
Unem. × 2008			0.314 (0.235)	0.180 (0.189)
Unem. × 2010			-0.111 (0.269)	0.125 (0.181)
Hausman test				
Log-lik	-4,393.05	-32,528.49	-4,091.06	-32,526.29
Observations	37,782	37,782	37,782	37,782

Coefficients statistically significant at the 10% level are in bold.

The estimation results for model M1 in Table 2 show that the information produced by the FE and the RE specifications is rather coherent with respect to the employment conditions: being employed with a temporary contract, being a first-job seeker or unemployed has a significant negative effect on the perceived health condition compared to being a permanent worker. In contrast, the coefficients related to the time dummies give different indications suggesting that, in the FE model, the occurrence of the economic downturn has damaged, albeit not significantly, the perceived health condition. The only difference in sign between the FE and RE models concerns the regional unemployment rate. The empirical evidence on the effect of the labor market context, such as local unemployment, on health is rather ambiguous. Our FE model shows a positive relationship between local unemployment and self-reported health, a result that has been found for some European countries as well [41]: being a temporary worker, unemployed or not employed is less stigmatizing in contexts where unemployment is common. In addition, the well-being of individuals disadvantaged in the labor market is known to decrease less if they live in environments with a high unemployment rate [42].

Finally, the *p*-value of the Hausman test (Table 2) leads us to reject the null hypothesis of correct specification of the RE assumptions, meaning that the results obtained by FE estimation are more reliable.

The columns labeled M2 in Table 2 refer to the estimation of ordered logit models where the latent health status is specified as


where *x*_*itj*_ are the dummy variables for the employment conditions, with *j*={Temporary, First-job seekers, Unemployed }, *t*=2008,2010, *A*_*t*_ are time dummies and the control variables have been omitted for brevity. This specification allows us to investigate whether the effect of the employment status on health has changed over time, possibly giving an indication of whether inequalities have strengthened with the occurrence of the economic crisis. However, the coefficients associated with the interaction terms *ϕ*_*tj*_ are not directly interpretable and further diagnostics are needed. In particular, we want to test whether the effect of being in a certain labor market condition in 2006, *β*_*j*_, is the same in 2008 or 2010. To this aim, we perform a Wald test for the null hypothesis *H*_0_:*δ*_*tj*_=0 where *δ*_*tj*_=*β*_*j*_-*γ*_*t*_-*ϕ*_*tj*_, which is equivalent to *H*_0_:*β*_*j*_=*γ*_*t*_+*ϕ*_*tj*_ and the value of test statistic is then compared with a  distribution. Results are displayed in Table 3: the inequality in the health status has widened between permanent workers and those looking for a job during the economic crisis. In particular, inequalities start to grow in 2008 with, however, only a short-term effect.

**Table 3:** **Diagnostic tests for time differences in self reported health status by employment status**

	***H***_0_:***β***_***j***_=***γ***_2008_+***ϕ***_2008,***j***_		***H***_0_:***β***_***j***_=***γ***_2010_+***ϕ***_2010,***j***_	
				
**Whole sample**				
Temporary	-0.347	0.307	-0.101	0.324
First-job seekers	**-0.566**	0.352	-0.343	0.377
Unemployed	**-0.624**	0.422	-0.243	0.468

Coefficients statistically significant at the 10% level are in bold.

As discussed above, there is extensive empirical evidence on differences in the relationship between the employment status and health between female and male workers. Gender inequalities in health are usually tied to the fact that females have a higher life expectancy than males. Moreover, our data suggest that the distribution of responses to SRHS by employment status is somewhat more uniform for women than for men^l^. Therefore, we estimate two separate models for men and women in order to detect such inequalities in the health status. Table 4 reports the estimation results obtained with the FE ordered logit model for the two sub-samples.

**Table 4:** **Fixed-effects ordered logit models for self reported health status by gender**

	Male		Female	
	M1	M2	M1	M2
	coeff. (s.e)	coeff. (s.e)	coeff. (s.e)	coeff. (s.e)
Ref: Permanent				
Temporary	**-0.288** (0.145)	**-0.331** (0.234)	0.080 (0.163)	0.159 (0.247)
First-job seekers	**-0.801** (0.208)	**-0.974** (0.278)	-0.052(0.220)	0.066 (0.314)
Unemployed	**-0.423** (0.212)	-0.371 (0.326)	-0.160 (0.245)	-0.456 (0.385)
2008	-0.001 (0.083)	-0.041 (0.092)	-0.013 (0.065)	-0.018 (0.071)
2010	0.140 (0.165)	0.171 (0.183)	-0.040 (0.128)	-0.026 (0.134)
Temp. × 2008		0.186 (0.265)		-0.119 (0.278)
Temp. × 2010		-0.114 (0.299)		-0.120 (0.291)
FJ seek. × 2008		**0.586** (0.289)		-0.316 (0.311)
FJ seek. × 2010		-0.071 (0.330)		0.011 (0.371)
Unem. × 2008		0.041 (0.342)		**0.911** (0.425)
Unem. × 2010		-0.115 (0.352)		-0.159 (0.457)
Hausman test	Rej.	Rej.	Rej.	Rej.
Log-lik	-1,985.44	-1,981.11	-2,005.71	-1,999.54
Observations	18,603	18,603	19,179	19,179

Coefficients statistically significant at the 10% level are in bold. Model specification also include the category for individuals out of the labor force, family income, family income square, family wealth, family wealth square, regional unemployment rate, age square. The Hausman test rejects the null hypothesis at the 5% level. Estimation results of the RE model are not presented here for brevity and are available upon request.

Male workers with a temporary contract, first-job seekers or unemployed present a significant decay in the health condition compared to permanent workers; however inequalities continue to grow until 2010 only for first-job seekers (see Table 5). In contrast, the health status of female workers does not seem to depend on the employment condition while a difference in the health condition emerges for unemployed women compared to permanent workers in 2008.

**Table 5:** **Diagnostic tests for time differences in self reported health status by employment status: gender**

	***H***_0_:***β***_***j***_=***γ***_2008_+***ϕ***_2008,***j***_		***H***_0_:***β***_***j***_=***γ***_2010_+***ϕ***_2010,***j***_	
				
**Male**				
Temporary	-0.476	0.449	-0.389	0.477
First-job seekers	**-1.501**	0.491	**-1.074**	0.527
Unemployed	-0.371	0.599	-0.427	0.646
**Female**				
Temporary	0.296	0.471	0.304	0.490
First-job seekers	0.400	0.557	0.080	0.604
Unemployed	**-1.349**	0.738	-0.271	0.768

Coefficients statistically significant at the 10% level are in bold.

Since our sample comprises individuals between 15 and 64 years of age, we also divide our sample in two age groups in order to investigate whether the relationship between employment and health differ across ages. Table 6 shows that only young individuals exhibit health differentials according to their working conditions. This has to be expected since young workers are those mainly targeted by a low degree of employment protection. Among the elders, only the unemployed show a decay in self-rated health compared to permanent workers. Moreover, the youngsters suffer from growing inequalities over time if they do not find a job (see Table 7).

**Table 6:** **Fixed-effects ordered logit models for self reported health status by age**

	15–40		41–64	
	M1	M2	M1	M2
	coeff. (s.e)	coeff. (s.e)	coeff. (s.e)	coeff. (s.e)
Ref: Permanent				
Temporary	**-0.211** (0.129)	**-0.313** (0.203)	-0.117 (0.171)	-0.041 (0.268)
First-job seekers	**-0.522** (0.162)	**-0.578** (0.218)	-0.694 (0.582)	-0.845 (0.833)
Unemployed	**-0.287** (0.210)	**-0.741** (0.356)	**-0.396** (0.216)	-0.169 (0.323)
2008	-0.030 (0.066)	**-0.107** (0.079)	-0.067 (0.057)	-0.063 (0.059)
2010	-0.018 (0.130)	-0.026 (0.145)	-0.104 (0.111)	-0.066 (0.114)
Temp. × 2008		0.173 (0.233)		0.002 (0.311)
Temp. × 2010		0.111 (0.252)		-0.252 (0.339)
FJ seek. × 2008		0.214 (0.224)		0.880 (0.846)
FJ seek. × 2010		-0.053 (0.253)		**-1.538** (1.10)
Unem. × 2008		**0.878** (0.382)		-0.150 (0.340)
Unem. × 2010		0.302 (0.401)		-0.383 (0.365)
Hausman test	Rej.	Rej.	Rej.	Rej.
Log-lik	-1,736.51	-1,731.90	-2,644.40	-2,641.23
Observations	17,422	17,422	20,360	20,360

See notes to Table 4.

**Table 7:** **Diagnostic tests for time differences in Self Reported Health Status by employment status: age**

	***H***_0_:***β***_***j***_=***γ***_2008_+***ϕ***_2008,***j***_		***H***_0_:***β***_***j***_=***γ***_2010_+***ϕ***_2010,***j***_	
				
**15-40**				
Temporary	-0.379	0.382	-0.398	0.407
First-job seekers	**-0.685**	0.376	**-0.499**	0.402
Unemployed	**-1.512**	0.673	**-1.017**	0.711
**41-64**				
Temporary	0.017	0.529	0.277	0.557
First-job seekers	-1.662	1.586	0.759	1.623
Unemployed	0.043	0.591	0.279	0.629

Coefficients statistically significant at the 10% level are in bold.

Finally, it is well known that the geographical location in Italy is tied to strong socio-economic inequalities, especially concerning labor market protection and opportunities. Moreover, since health care expenditure is in hands of the regional governments, differences in health care emerge across the country, where the north benefits from a more efficient health system compared to the rest of Italy [28]. Therefore, we finally investigate whether there is a territorial heterogeneity in the effect of the employment condition on the perceived health status. Descriptive statistics already suggest that there is a strong dichotomy between the north and the rest of Italy in terms of self-assessed health conditions (see Table 1).

Table 8 reports the estimation results of the FE models by area of residence, that is the north of Italy and the rest of the territory. We have to consider the center and south of Italy together because of quasi-collinearity issues; moreover Table 1 shows that their health patterns are indeed similar. We find evidence that the category of first-job seekers experience a worse health condition in the north of Italy, while all profiles exhibit a significant lower health condition compared to permanent workers in the center and south of Italy.

**Table 8:** **Fixed-effects ordered logit models for self reported health status by area of residence**

	North		Rest of Italy	
	M1	M2	M1	M2
	coeff. (s.e.)	coeff. (s.e.)	coeff. (s.e.)	coeff. (s.e.)
Ref: Permanent				
Temporary	-0.090 (0.168)	0.046 (0.253)	**-0.172** (0.128)	**-0.334** (0.204)
First-job seekers	**-0.561** (0.306)	-0.589 (0.501)	**-0.448** (0.163)	**-0.586** (0.218)
Unemployed	-0.220 (0.287)	**-1.041** (0.566)	**-0.347** (0.180)	-0.323 (0.276)
2008	**0.200** (0.067)	**0.213** (0.072)	**-0.223** (0.057)	**-0.295** (0.065)
2010	0.144 (0.193)	0.154 (0.197)	-0.065 (0.097)	-0.056 (0.104)
Temp. × 2008		-0.329 (0.299)		**0.380** (0.232)
Temp. × 2010		-0.069 (0.317)		0.057 (0.251)
FJ seek × 2008		-0.042 (0.588)		**0.396** (0.222)
FJ seek × 2010		0.094 (0.617)		0.000 (0.252)
Unem. × 2008		**0.892** (0.648)		0.322 (0.289)
Unem. × 2010		**1.035** (0.624)		-0.275 (0.308)
Hausman test	Rej.	Rej.	Rej.	Rej.
Log-lik	-1,865.19	-1,715.23	-2,504.54	-2,497.55
Observations	16,294	16,294	21,488	21,488

See notes to Table 4.

For temporary workers in the north of Italy, health inequalities do not arise over time while they grow for the unemployed (see Table 9). In the rest of Italy, differences in the perceived health condition widen over time for those looking for a job for the first time.

**Table 9:** **Diagnostic tests for time differences in self reported health status by employment status: area of residence**

	***H***_0_:***β***_***j***_=***γ***_2008_+***ϕ***_2008,***j***_		***H***_0_:***β***_***j***_=***γ***_2010_+***ϕ***_2010,***j***_	
				
**North**				
Temporary	0.163	0.495	-0.039	0.5264
First-job seekers	-0.757	1.005	-0.834	1.049
Unemployed	**-2.145**	1.149	**-2.229**	1.147
**Rest of Italy**				
Temporary	-0.420	0.392	-0.335	0.413
First-job seekers	**-0.686**	0.380	**-0.530**	0.409
Unemployed	-0.349	0.503	0.008	0.538

Coefficients statistically significant at the 10% level are in bold.

## Conclusions

The incidence of non-standard labor contracts on permanent employment has been steadily increasing and job opportunities for the unemployed and first–job seekers have drastically diminished in the last decade. In this framework, work arrangements and job deprivation have recently become the focus of many empirical contributions aimed at assessing whether an increasing degree of job insecurity and lack of job opportunities are related to worse physical and mental health.

The results of our empirical analysis confirm that temporary workers, first-job seekers and unemployed individuals all experience a worse health conditions than permanent workers. Moreover, differentials in the health status spread in times of economic strain for those looking for job opportunities. Although Italian women are most likely targeted by the absence of employment protection (49.9% of working women was employed with a temporary contract in 2010) and are those with a higher unemployment rate [1], we find that economic inequalities among female workers and job-seekers do not reflect onto their reported health condition. On the contrary, strong inequalities in the health outcome emerge among men and grow over time for those looking for their first job opportunity. Young Italian temporary workers and job seekers^m^ experience a strong decay in health compared to their permanently employed peers which grows over time for those looking for a job after 2006. Finally, there is a remarkable difference in health differentials between the north and the rest of the country where health deteriorates for all employment profiles compared to permanent employment. However, after the occurrence of the economic crisis, inequalities between permanent workers and the unemployed arise in the north of the country as well.

The question addressed in this paper is particularly relevant in the context of a dual labor market: in Italy a strong dichotomy has emerged, where on one side employees with open–ended contracts enjoy the benefits of a high degree of protection, while on the other temporary workers are exposed to high job insecurity and a very low, if none, degree of employment protection; moreover, welfare is not always able to adequately sustain workers during spells of unemployment, whose health condition may deteriorate due to the economic deprivation. The financial situation of the country, especially with the occurrence of the recent economic downturn, has led to substantial cuts in public expenditure and also in health care, where an average yearly decrease of -0.4*%* of GDP has been registered only from 2009 to 2011, compared to the average 0.2*%* of OECD countries [43]. In this context, growing inequalities in the health condition between economically deprived categories and financially stable ones would, in contrast, call for a stronger presence of the public health care system.

## Endnotes

^a^ The literature on unemployment and health is extensive. For brevity, we mention only the most recent contributions here and refer the reader to the cited papers for more extensive literature reviews.

^b^ See [44] for further details.

^c^ For more details on the survey design, means of collections and response rates see [45] and the website https://www.bancaditalia.it/statistiche/indcamp/bilfait.

^d^ It could be argued that transitions in and out of the various employment profiles should be modeled instead. However, breaking our profiles further apart results in too few observations for each category leading to quasi-collinearity problems since we model all possible labor market conditions.

^e^ The information on the working status is provided by the variable APQUAL in the SHIW questionnaire.

^f^ For dependent workers, there is a specific category of APQUAL that isolates non-standard contracts. For the self-employed, we cross-reference the categories in APQUAL with the variable CONTRATT, that takes values 2 and 3 if the job is not permanent. Within the *Temporary* category, individuals could be divided into fixed-term workers (who benefit from more unemployment protection) and seasonal/on-call workers. However, this last category identifies only 232 individuals and cannot be considered separately in the estimation without incurring in severe convergence problems.

^g^ Hypothetically, someone whose illness led him/her to stop working because unable to cope with the demands of the job could actively be looking for another job and therefore result as unemployed. However, such an event is very rare in Italy, where the labor law allows ill workers to suspend their contract even for long periods (from 180 days to 2 years). In addition, they cannot be dismissed while the contract is suspended and are entitled to an allowance from social insurance that covers, on average, 2/3 of their salary during the first 180 days of sick leave. See http://www.ilo.org/global/lang--en/index.htmfor further details.

^h^ Since it is computed using both dependent labor income and self-employment, income can be negative.

^i^ We matched data on the regional unemployment rate published by the Italian Institute of Statistics with SHIW data at the regional level.

^j^ See [37] and [38] for further details.

^k^ See [46] for a detailed illustration of the estimation procedure.

^l^ About 55% of responses in sample of female is *good* (the modal value) in any employment category (except for the unemployed with 53%) while the percentage of responses to the *good* category in the male sample (the modal value) is decreasing with the employment condition: 55% of *permanent*, 53% of *temporary*, 52% of *unemployed*, and 46% of *inactive*.

^m^ 27.8% aged 15-24 compared to 3.6% aged 55-64 in 2010 [1].

## Abbreviations

SRHS: Self reported health status
SHIW: Survey on household income and wealth.
